# Follicular dendritic cell sarcoma involving the parotid gland with expression of the melanocytic marker PRAME

**DOI:** 10.1007/s12308-024-00605-7

**Published:** 2024-10-09

**Authors:** Sumayya Aslam, Ifegwu Ibe, Ying Zhang, Roksolana Demianets, Truc Tran, Ashley Gamayo, Xiaohui Zhao, Sherif A. Rezk

**Affiliations:** Department of Pathology and Laboratory Medicine, Irvine Medical Center (UCI), University of California, 101 The City Drive, Bldg. 54, Rm 4702, Orange, CA 92868 USA

**Keywords:** Follicular dendritic cells, Sarcoma, Parotid gland, PRAME, Melanoma

## Abstract

Follicular dendritic cell sarcoma is a rare mesenchymal neoplasm arising from follicular dendritic cells (FDC) of lymphoid follicles. While the majority of FDC sarcoma cases arise within lymph nodes, approximately 30% manifest in extranodal sites. Only 4 prior occurrences of intra-parotid FDC sarcomas have been documented. We are reporting a rare case of FDC of the parotid gland in a 65-year-old male with a questionable history of B-cell lymphoma. The patient underwent a right total parotidectomy and bilateral neck dissection. A diagnosis of follicular dendritic cell (FDC) sarcoma was made, with one positive intra-parotid node. The malignant cells expressed the characteristic markers for FDC sarcoma but with positivity of the melanocytic marker PRAME. This is a case of FDC sarcoma with an unusual extranodal localization in the parotid gland. Immunohistochemistry was useful in making a diagnosis although the positivity for the melanocytic marker PRAME was unusual and unreported before.

## Introduction

Soft tissue sarcomas are rare tumors comprising roughly 1% of malignancies in adults. Despite their rarity, they exhibit a substantial mortality rate, contributing to about 3–4% of cancer-related fatalities each year [1]. FDC sarcoma is an exceedingly uncommon form of sarcoma characterized by its low to intermediate malignant nature. It originates from follicular dendritic cells, yet instances of its occurrence in extranodal locations such as the mediastinum, gastrointestinal tract, liver, and spleen have also been documented [1–3]. Only four prior occurrences of intra-parotid FDC sarcomas have been documented [2].

## Clinical history

Our patient is a 65-year-old male who presented with a right parotid mass and bilateral neck lymphadenopathy. He had an undocumented history of a cutaneous right cheek lesion that was previously biopsied and thought to represent B-cell lymphoma. On examination, there was a firm mass at the right parotid tail. There was also a palpable right neck lymphadenopathy in level 2A and level 3. Magnetic resonance imaging (MRI) of the neck showed a 5.1 × 4.5 × 8.3 cm enhancing heterogeneous T2 hyperintense lesion involving the right superficial parotid gland.

## Results

**Fig. 1:**
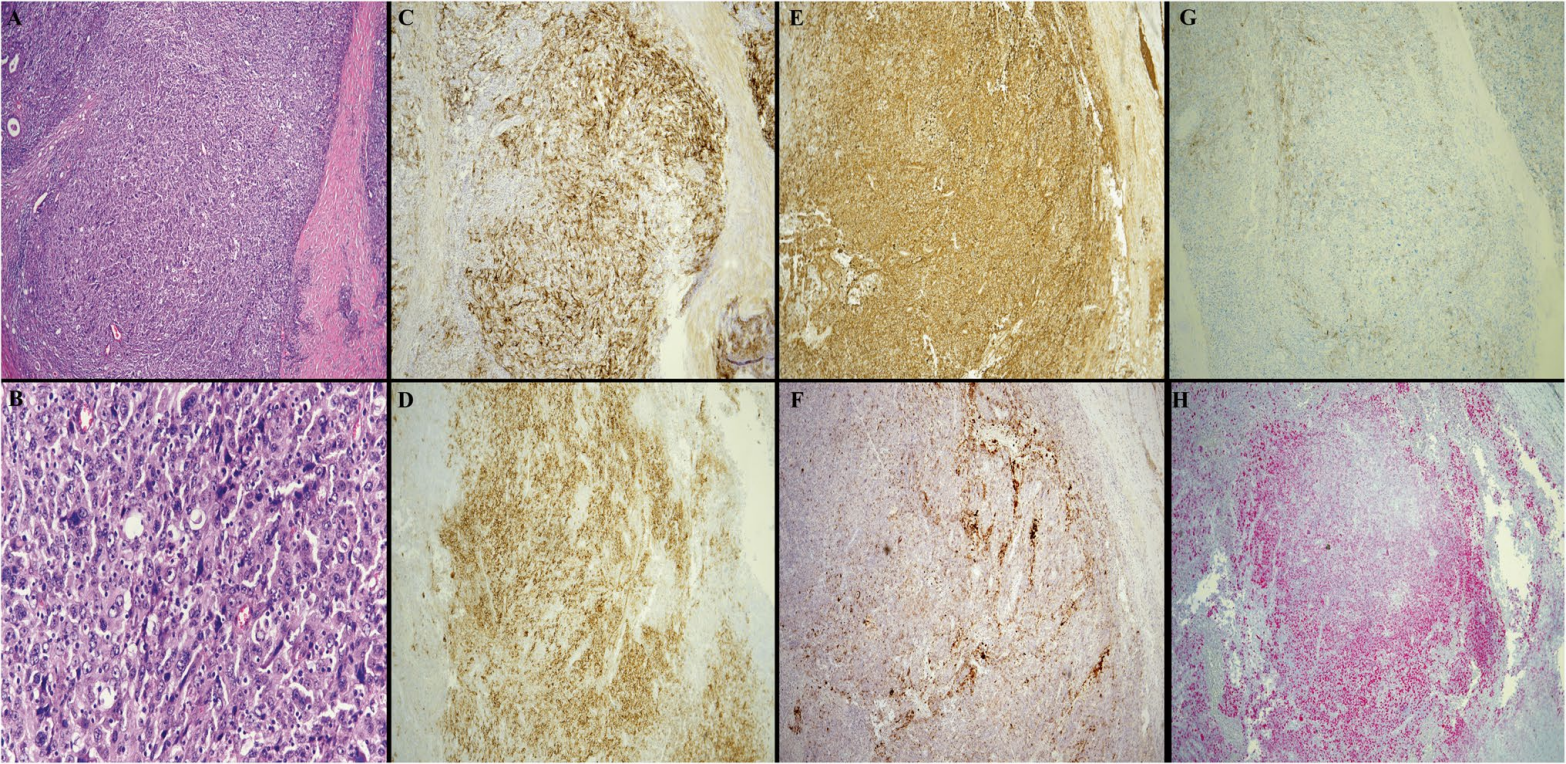
The composite picture represents parotid gland involvement by follicular dendritic cell sarcoma. **A** H&E stain, low-power magnification (4 ×) of the sarcoma cells with rare unremarkable glands noted. **B** H&E stain, high-power magnification (40 ×) showing the large sarcoma cells, mostly with one centrally placed nucleoli and open chromatin. Scattered large highly atypical malignant cells are noted. Few interspersed small lymphocytes are also seen. **C** CD21 expression by the sarcoma cells (4 ×). **D** CD23 expression (4 ×). **E** Clusterin expression (4 ×). **F** CXCL13 expression (4 ×). **G** CD35 partial expression (4 ×). **H** PRAME expression (4 ×)

Initially, an ultrasound-guided core biopsy was performed, which showed a poorly differentiated neoplasm, suggestive of FDC sarcoma. The patient then underwent right total parotidectomy and bilateral neck dissection. Sections of the parotid mass showed an infiltration of large cells with irregular nuclei, vesicular chromatin, prominent nucleoli, and moderate cytoplasm. A subset of the cells showed atypia with enlarged, highly irregular, and hyperchromatic nuclei. The malignant cells expressed CD21, CD23 (subset), CD35 (small subset), CXCL13 (subset), vimentin, fascin, and clusterin, suggestive of FDC origin (Fig. 1). The malignant cells also expressed CD4 and CD5 (subset) but were negative for all other T-cell markers (CD2, CD3, CD7, CD8, CD43, TIA-1, BF-1). Since a subset of FDC sarcomas can be associated with indolent T-lymphoblastic proliferations, TdT stain was performed and is negative. EBV was negative by in situ hybridization (EBER). Podoplanin (D2-40), which can be utilized as a marker for follicular dendritic cells was negative in our case. In addition, the malignant cells were positive for PRAME but negative for all other melanoma markers (S100, HMB45, Melan A, and SOX10). The infiltrate involved the parotid gland parenchyma and directly adjacent lymph nodes. Table 1 illustrates the different antibody clones used in the case. A next-generation sequencing (NGS) test was performed (Tempus 648 genes xT panel) and it detected 8 likely pathogenic somatic variants, including TP53, RB1, and FBXW7 loss-of-function variants. B-cell gene rearrangement studies by polymerase chain reaction (PCR) were performed but showed inconclusive results. Table 2 illustrates the different mutations detected along with their variant allele frequency (VAF). Taken together, the overall picture supports a diagnosis of follicular dendritic cell (FDC) sarcoma. A follow-up appointment was arranged with the Radiation Oncology department for further assessment and management.

**Table 1:** List of immunohistochemical markers used and their antibody clones

Immunohistochemical stains	Antibody clones
BF-1	Invitrogen (8A3)
CD2	Cell Marque (MRQ-11)
CD3	Roche (2GV6)
CD4	Cell Marque (SP35)
CD5	Roche (SP19)
CD7	Cell Marque (MRQ-56)
CD8	Cell Marque (C8/144B)
CD21	Dako (1F8)
CD23	Roche (SP23)
CD35	Dako (Ber-MAC-DRC)
CD43	Roche (L60)
CD45	Agilent (2B11 + PD7/26)
CD68	Roche (KP-1)
Anti-Clusterin	Millipore (41D)
CXCL 13	R & D Systems (53610)
EMA	Cell Marque (E29)
Fascin	Cell Marque 55 K-2
HMB-45	Cell Marque (HMB-45)
Melan-A	Roche (A103)
Podoplanin	Cell Marque (D2-40)
PRAME	Abcam (EPR20330)
S100	Roche (Polyclonal)
SOX-10	Cell Marque (EP268)
Tia-1	Biocare (TIA1)
Tdt	Agilent (EP266)
Vimentin	Roche (V9)

**Table 2:** Summary of tumor genomic profiles of our case by NGS

Tumor-specific gene mutation	Variant allele fraction (VAF)
TP53: c.560-1_560delinsAA, p.G187D, NM_000546	22.2%
ATR: c.481A > T p.R161*, NM_001184	21.6%
KDM6A: c.571C > T p.Q191*, NM_001291415	21.4%
TERT: c.-146C > T, NM_198253	20.1%
RB1: c.1547G > A p.W516*, NM_000321	17.2%
FBXW7: c.881del p. S294fs, NM_033632	12.5%
NOTCH1: c.1255 + 1G > A, NM_017617	10.1%
FHIT: c.250-1G > A, NM_001166243	8.1%

## Discussion

Follicular dendritic cells are a specialized type of dendritic cells that are largely restricted to lymphoid follicles. They form dense three-dimensional meshworks within benign follicles, which maintain the follicular architecture [4]. FDC sarcoma is a neoplastic proliferation of cells showing morphologic and immunophenotypic features of follicular dendritic cells [5]. The etiology of that neoplastic transformation is unknown although it may evolve in situations in which there is FDC hyperplasia and overgrowth [4]. It usually occurs de novo; however, it can sometimes occur in association with hyaline vascular Castleman disease, whether simultaneously or as a succeeding event [4]. It presents as a painless solid mass, usually nodal (mainly cervical lymph nodes) but it can also involve extra nodal sites, such as tonsils, spleen, skin, and gastrointestinal tract [4, 6, 7]. A new variant has been recently described: EBV-positive inflammatory follicular dendritic cell tumor and is reported to occur exclusively in the liver and spleen, exhibit more interspersed lymphoplasmacytic infiltrate, and express EBV by in situ hybridization [8]. Overall, FDC sarcoma is considered a low-grade sarcoma that has a significant recurrence rate in nearly half the cases, and it also can metastasize [3]. Surgical resection remains the best treatment for these tumors.

Histologically, these tumors can be difficult to diagnose, as the morphological spectrum is broad and often causes confusion. Cytological atypia is present only in a subset of cases and mitotic figures are common but highly variable in number. By immunohistochemistry, FDCs express CD21, CD23, CD35, CXCL13, and clusterin. They also usually express vimentin, fascin, HLA-DR, and EMA and variably positive for CD68, S100, and CD45 [1, 4]. Clusterin staining is reported to be highly sensitive (100%) and specific (93%) and along with CD21 and CD23, constitute the essential stains required to establish a definitive diagnosis [9]. PRAME stain exhibits diffuse positivity in most melanomas, while typically presenting as negative or showing limited and focal immunoreactivity in nevi [10]. Variable degrees of PRAME staining have been sporadically observed in other malignant tumors, including most synovial sarcomas, myxoid liposarcomas, and malignant peripheral nerve sheath tumors (MPNST) [10]. Other neoplasms such as seminomas and carcinomas of various origins including endometrial, serous ovarian, mammary ductal, lung, and renal showed an intermediate proportion of cases and variable extent of tumor cells positive for PRAME protein expression [10]. To our knowledge, PRAME positivity has not been reported in FDC sarcoma before. In our case, PRAME is positive but all other melanoma markers (S100, HMB45, Melan A, and SOX10) are negative. Few FDC sarcoma cases with aberrant phenotype have been reported before including a case of intra-abdominal FDC sarcoma with pleomorphic features and aberrant expression of neuroendocrine markers [11], an unusual case of FDC sarcoma of the omentum with pleomorphic morphology and aberrant cytokeratin expression [12], another case with aberrant T-cell antigen expression [13], and a clinicopathologic study of 15 FDC cases with expression of MDM2, somatostatin receptor 2A, and PD-L1 [14].

Although genetic drivers for tumorigenesis in FDC are largely unknown, recent genomic profiling studies have revealed several recurrent gene alterations in FDC sarcoma, including BRAF V600E mutation [15] and loss-of-function variants in tumor suppressor genes involved in the regulation of NF-κB pathway and cell cycle, such as NFKBIA, CYLD, CDKN2A, and RB1 genes [16]. In addition, genomic profiling for one patient with primary esophageal follicular dendritic cell sarcoma revealed pathogenic variants in multiple genes, including CHEK2, FAT1, TP53, DPYD, ERBB2IP, FBXW7, KMT2D, PPP2R1A, and TSC2 [17]. The NGS results for this patient identified loss-of-function pathogenic variants in RB1 (p.W516*), TP53 (p.G187D), and FBXW7 (p.S294fs), which have been reported previously in FDC sarcoma patients, supporting the FDC sarcoma diagnosis.

In conclusion, we report a case of FDC sarcoma with an unusual extranodal localization in the parotid gland. Furthermore, the aberrant positive expression of the melanocytic marker PRAME has not been reported before. All other melanocytic markers were negative in our case and the characteristic FDC markers are positive.
